# Oral Health‐Related Quality of Life, Perceived Stress, and Well‐Being in Oral Lichen Planus Subtypes

**DOI:** 10.1002/cre2.70468

**Published:** 2026-11-09

**Authors:** Kim N. Stolte, Sumru Sinem Dogan, Sameh Attia, Henrik Dommisch

**Affiliations:** ^1^ Department of Periodontology Oral Medicine and Oral Surgery, Charité—Universitätsmedizin Berlin Berlin Germany

**Keywords:** oral lichen planus, patient‐reported outcome measures, quality of life

## Abstract

**Objective:**

Oral lichen planus (OLP) is a chronic inflammatory mucosal disease with reticular and erosive clinical subtypes. This study aimed to compare oral health‐related quality of life (OHRQoL), stress, and general well‐being between participants with reticular OLP, erosive OLP, and healthy controls.

**Material and Methods:**

In this single‐center cross‐sectional study, healthy controls (*n* = 20) and patients with reticular (*n* = 20) or erosive OLP (*n* = 20) completed the OHIP‐G14, PSS‐10, and WHO‐5‐questionnaires, and group differences were evaluated.

**Results:**

Patients with OLP reported significantly poorer OHRQoL than controls (OHIP‐G14 total score: 13.18 ± 10.01 vs. 2.85 ± 6.54; *p* < 0.001). Significant group differences were observed across all OHIP‐G14 subdomains. Erosive OLP showed higher scores than reticular OLP across domains, though subtype differences were not significant. In both OLP groups, the highest mean scores were observed for physical pain and psychological discomfort. No significant between‐group differences were observed for PSS‐10 or WHO‐5 (all *p* ≥ 0.259). Interpretation is limited by differences in baseline characteristics between groups.

**Conclusion:**

Patients with OLP reported poorer OHRQoL than healthy controls, whereas differences between reticular and erosive OLP were not significant. The findings suggest that oral‐specific and generic patient‐reported outcome measures capture complementary aspects of patient experience, with OHIP‐G14 reflecting oral disease‐specific burden in OLP.

## 1 Introduction

Oral lichen planus (OLP) is a chronic immune‐mediated inflammatory disease of the oral mucosa and is classified by the World Health Organization as an oral potentially malignant disorder with heterogeneous clinical presentation (Warnakulasuriya et al. 2007, 2021). Divided into six clinical forms by Andreasen, the most common subtypes are the reticular form and the erosive variants that are frequently associated with burning, pain, and functional impairment (Andreasen 1968). The burden of OLP extends beyond clinician‐rated lesion severity, as symptoms and restrictions in daily life can vary substantially among patients with comparable clinical findings. Reliance on clinical severity scoring alone may therefore underestimate patient‐perceived burden and fail to capture clinically relevant effects on daily functioning (Yiemstan et al. 2020). Consequently, patient‐reported outcomes (PROs) have become increasingly important for assessing the impact of oral diseases from the patient perspective and for complementing clinical endpoints in clinical research. This development is also reflected in periodontal research, where quality of life has been defined as a mandatory outcome domain in a core outcome set for periodontal trials (Lamont et al. 2021). Recent studies further suggest that clinician‐rated and patient‐reported burden in OLP are related but not interchangeable, underlining the importance of PROs as complementary outcomes in both clinical care and research (Myers et al. 2026). In addition, a dedicated review of PRO use in OLP has highlighted substantial heterogeneity in the instruments used and emphasized the need for robust and interpretable measures tailored to this population (Wiriyakijja et al. 2018).

Oral health‐related quality of life (OHRQoL) instruments quantify the extent to which oral conditions affect physical, psychological, and social functioning. The Oral Health Impact Profile‐14 (OHIP‐14), a widely used short form instrument derived from the original OHIP, provides a multidimensional assessment of oral impacts in everyday life (Slade 1997). In OLP, both individual studies and meta‐analytic evidence indicate that OHRQoL is impaired compared with healthy controls and that OHIP scores correlate with clinical severity (Yuwanati et al. 2021; Stolte et al. 2024). This association is clinically relevant because validated OHRQoL instruments can capture treatment‐relevant impairment that may not be fully reflected by lesion morphology alone (Yiemstan et al. 2020). However, despite the growing body of literature on OHRQoL in OLP, direct comparisons across clinically defined subtypes using consistent PROs remain valuable, particularly when paired with patient‐centered questionnaires addressing psychological dimensions. Such an approach may help clarify whether observed impairments are predominantly oral‐condition‐specific or instead reflect broader psychosocial sequelae. Reported psychological correlates in the literature are heterogeneous, providing a rationale for simultaneous assessment of oral‐specific and generic PRO constructs (Cerqueira et al. 2018; Radwan‐Oczko et al. 2018).

Therefore, the aim of the present study was to identify differences in OHRQoL, stress, and well‐being between reticular and erosive OLP when compared to healthy controls. We hypothesized that participants with erosive OLP would report poorer OHRQoL than those with reticular OLP and healthy controls.

## 2 Materials and Methods

### 2.1 Study Design and Participants

This single‐center cross‐sectional comparative study was conducted at Charité—Universitätsmedizin Berlin, Germany, and 60 participants were included. Recruitment and data collection took place between January 2024 and July 2025. Owing to limited prior evidence on direct comparisons of patient‐reported outcomes across clinically defined OLP subtypes, this study was designed as an exploratory pilot study intended to generate hypotheses rather than confirmatory evidence. Equally sized groups of healthy controls (*n =* 20), patients with reticular OLP (*n =* 20), and patients with erosive OLP (*n =* 20) were included. Participants were not matched for age, sex, or comorbidities. Baseline demographic and clinical characteristics were summarized descriptively for each study group (Table 1).

**Table 1:** Descriptive characteristics of the study groups.

	Control group	Reticular OLP	Erosive OLP
Sex			
Female	60%	70%	75%
Male	40%	30%	25%
Age in years	33.45 ± 14.19	60.00 ± 10.55	62.96 ± 8.06
Thyroid dysfunction	0%	40%	20%
Hypertension	15%	45%	55%
Diabetes mellitus	5%	0%	10%
Antidepressant medication	5%	10%	0%
Self‐reported gum bleeding	35%	50%	80%
Frequently	5%	10%	20%
Rarely	30%	40%	60%
Never	65%	50%	20%
Smoking	15%	10%	0%

### 2.2 Eligibility Criteria and Participant Recruitment

All participants were required to meet the following inclusion criteria: age 18–85 years and ability to provide written informed consent. OLP diagnosis was based on both clinical criteria (Andreasen 1968) and histopathological features (van der Meij and van der Waal 2003). Clinical phenotyping of OLP was performed by a specialized oral surgeon (K.N.S.) based on clinical presentation, with histopathological confirmation. Controls had no current or previous diagnosis of OLP and were required to have clinically healthy oral mucosa without signs of inflammatory oral mucosal disease.

Participants were excluded if any of the following criteria applied: pregnancy or breastfeeding, current or previous oral carcinoma, current drug therapy for OLP, alcohol dependence, terminal or severe systemic disease with marked impairment of general vitality, chemotherapy, radiotherapy, or inability to provide informed consent for any reason.

### 2.3 Patient‐Related Measures

Oral health‐related quality of life was assessed using the German 14‐item short form of the Oral Health Impact Profile‐14 (OHIP‐G14), which captures impacts across seven domains: functional limitation, physical pain, psychological discomfort, physical disability, psychological disability, social disability, and handicap. The instrument yields both subdomain scores and a total score (Slade 1997; John et al. 2006). Scores were calculated as sum scores according to the instrument manual, with higher scores indicating poorer OHRQoL.

Perceived stress was measured using the German adaptation of the 10‐item Perceived Stress Scale (PSS‐10), which assesses perceived stress over the preceding month (Cohen et al. 1983; Schneider et al. 2020). The questionnaire uses a 1‐5 Likert response scale (1 = never, 5 = very often). Perceived helplessness (PSS‐H; items 1, 2, 3, 6, 9, and 10) and perceived self‐efficacy (PSS‐S; items 4, 5, 7, and 8) were calculated as the sums of their respective items. Total PSS‐10 scores were calculated by reverse‐scoring PSS‐S items and summing all 10 items, yielding a possible score range of 10–50. Higher scores indicate greater perceived stress.

General well‐being was assessed using the WHO‐5 Well‐Being Index, a brief instrument and one of the most widely used questionnaires for the assessment of current subjective psychological well‐being (Heun et al. 2001; Topp et al. 2015). WHO‐5 scores were calculated according to the standard scoring procedure, with higher scores indicating better well‐being. Patient‐reported outcomes were treated as continuous variables. No missing questionnaire data occurred.

### 2.4 Statistical Analyses

Baseline demographic and clinical characteristics were summarized descriptively for each study group. Group differences in patient‐reported outcomes were assessed across the three study groups (healthy controls, reticular OLP, erosive OLP). Normality was evaluated using the Shapiro–Wilk test. Depending on distributional characteristics of the data, one‐way ANOVA or the Kruskal–Wallis test was applied, as appropriate. For significant overall group effects, post‐hoc pairwise comparisons with Bonferroni adjustment were performed. A *p*‐value of < 0.05 was considered statistically significant. Descriptive statistics are presented as mean value and standard deviation (SD). Statistical analyses were conducted using IBM SPSS Statistics (version 30.0.0.0).

## 3 Results

### 3.1 Sample Characteristics

The study groups comprised healthy controls, patients with reticular OLP, and patients with erosive OLP (Figure 1). The study included 60 participants with an average age of 52.13 ± 17.34 years, and 68.3% of participants were female. Healthy controls were younger than patients with reticular and erosive OLP and showed a lower prevalence of several comorbidities, including hypertension and thyroid dysfunction (Table 1). These baseline differences were taken into account when interpreting comparisons between groups.

**Figure 1:**
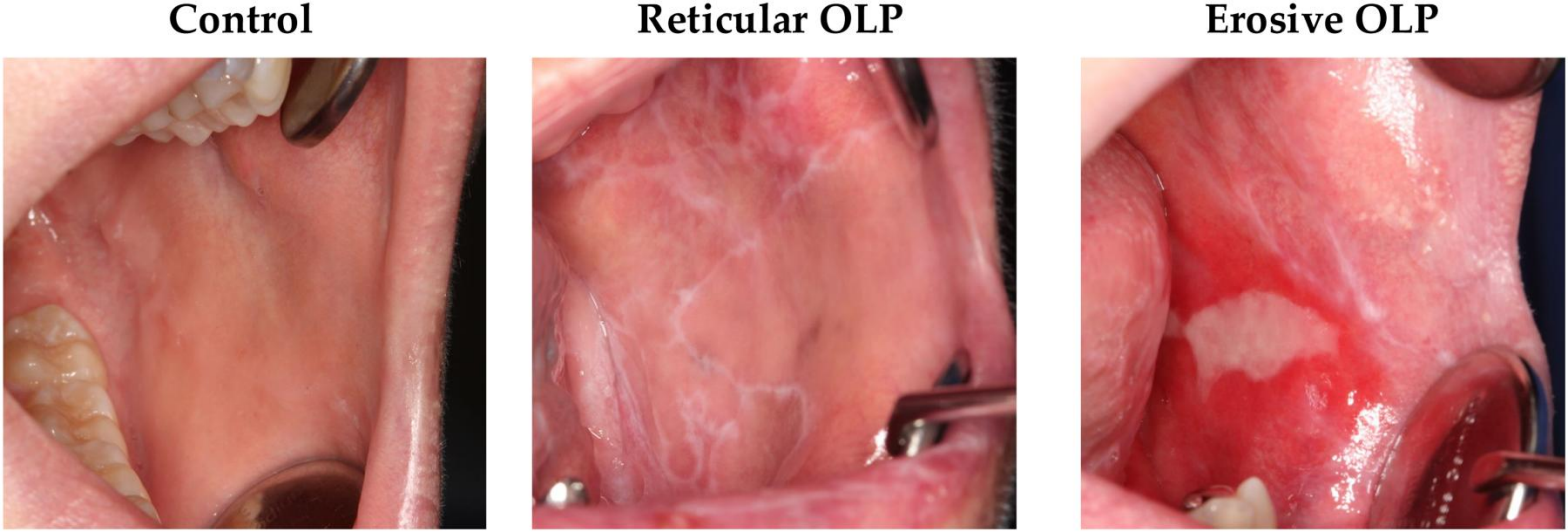
Representative clinical presentation of the study groups.

Representative clinical photographs of the buccal mucosa are shown for healthy controls, reticular OLP, and erosive OLP.

### 3.2 Oral Health‐Related Quality of Life (OHIP‐G14)

In unadjusted comparisons, patients with OLP reported significantly higher OHIP‐G14 total scores than healthy controls (13.18 ± 10.01 vs. 2.85 ± 6.54; *p* < 0.001; Table S1), indicating impaired OHRQoL (Figure S1). Total OHIP scores differed significantly between controls and both the reticular subtype (*p* = 0.003) and the erosive subtype (*p* = 0.000, Figure 2).

**Figure 2:**
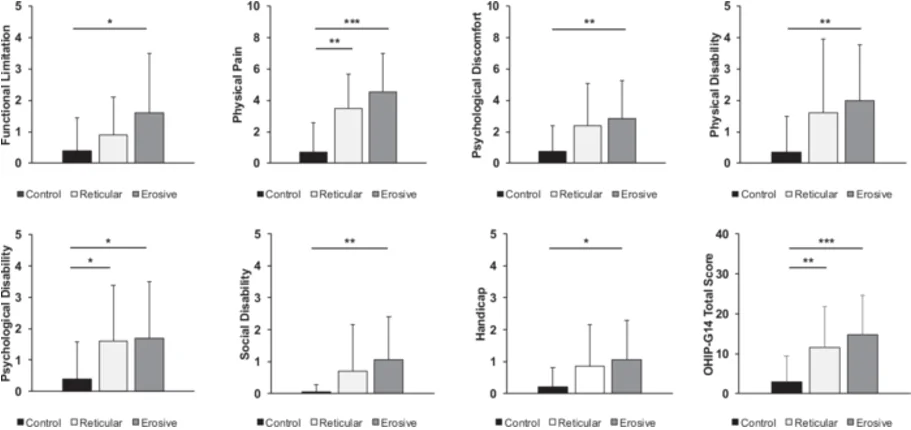
Oral Health Impact Profile‐14 (OHIP‐G14) outcomes in healthy controls and patients with reticular and erosive OLP. Bars represent mean values with standard deviation for each OHIP‐G14 subdomain and the total OHIP‐G14 score. Asterisks indicate significant Bonferroni‐adjusted pairwise comparisons (*p < 0.05, **p < 0.01, ***p < 0.001).

Across all OHIP‐G14 subdomains, the erosive subtype demonstrated higher mean scores than the reticular subtype; however, differences between the two OLP subtypes were not significant. In both OLP groups, the highest mean scores were observed in the domains of physical pain, followed by psychological discomfort (Table 2).

**Table 2:** OHIP‐G14 scores: Overall group differences (mean value ± SD) among healthy controls, patients with reticular OLP, and patients with erosive OLP.

OHIP‐G14 subdomain	Control	Reticular OLP	Erosive OLP	*p*‐value
Functional limitation	0.40 ± 1.05	0.90 ± 1.21	1.60 ± 1.90	0.036
Physical pain	0.70 ± 1.90	3.50 ± 2.19	4.55 ± 2.44	< 0.001
Psychological discomfort	0.75 ± 1.65	2.40 ± 2.68	2.85 ± 2.43	0.007
Physical disability	0.35 ± 1.14	1.60 ± 2.35	2.00 ± 1.78	0.002
Psychological disability	0.40 ± 1.19	1.60 ± 1.79	1.70 ± 1.81	0.007
Social disability	0.05 ± 0.22	0.70 ± 1.46	1.05 ± 1.36	0.007
Handicap	0.20 ± 0.62	0.85 ± 1.31	1.05 ± 1.23	0.021
Total OHIP‐G14 score	2.85 ± 6.54	11.55 ± 10.15	14.80 ± 9.86	< 0.001

While the reticular subtype showed significant differences relative to healthy controls exclusively in the domains of physical pain (*p* = 0.001) and psychological disability (*p* = 0.024), the erosive subtype demonstrated significant impairments across all OHIP‐G14 subdomains (*p* ≤ 0.030).

### 3.3 Perceived Stress Scale (PSS‐10)

Perceived stress did not differ significantly between healthy controls and patients with OLP (Figure 3A–C and Table S2). Mean PSS total scores were comparable between groups (healthy, 12.50 ± 5.58; reticular, 14.60 ± 6.30; erosive, 12.50 ± 6.18; *p* = 0.450). The same pattern was observed for the PSS subdomains. Scores for the helplessness subscale, reflecting perceived lack of control/helplessness (PSS‐H), were similar across groups (healthy, 13.90 ± 4.23; reticular, 14.65 ± 4.74; erosive, 12.60 ± 4.20; *p* = 0.335). Likewise, no significant differences were found for the self‐efficacy subscale, which reflects perceived coping/self‐efficacy (PSS‐S; healthy, 8.60 ± 3.56; reticular, 9.95 ± 2.70; erosive, 9.90 ± 3.96; *p* = 0.185).

**Figure 3:**
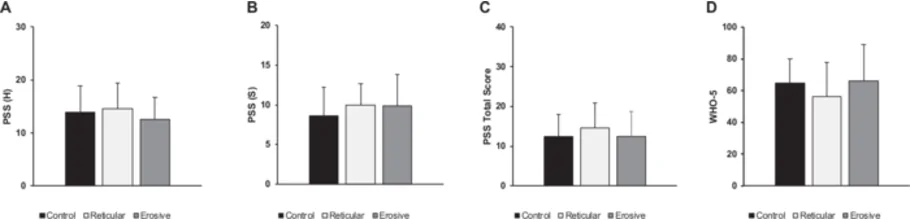
Perceived Stress Scale (PSS‐10) and WHO‐5 well‐being scores in healthy controls and patients with reticular and erosive OLP. Bars show mean value and standard deviation for the PSS helplessness subscale (PSS‐H; A), the PSS self‐efficacy subscale (PSS‐S; B), the PSS total score (C) and standardized WHO‐5 well‐being score (D) in healthy controls and patients with reticular and erosive OLP.

### 3.4 General Well‐Being (WHO‐5)

General well‐being, as measured by the WHO‐5 questionnaire, did not differ significantly between healthy controls and patients with OLP (*p* = 0.259, Figure 3D). Healthy controls had a mean score of 16.15 ± 3.82 (64.60% ±15.28), patients with reticular OLP had a mean score of 14.05 ± 5.38 (56.20% ± 21.52) and patients with erosive had a mean score of 16.50 ± 5.72 (66.00% ± 22.88). Using the commonly applied WHO‐5 screening threshold (raw score < 13), 4/20 (20%) healthy controls, 6/20 (30%) participants with reticular OLP, and 4/20 (20%) participants with erosive OLP scored below the threshold, with similar proportions across the three study groups.

## 4 Discussion

In this exploratory cross‐sectional comparative study, participants with OLP reported substantially poorer OHRQoL than healthy controls, whereas perceived stress and general well‐being did not differ significantly between groups. These findings indicate pronounced unadjusted differences in oral health‐related burden within the present cohort. However, age imbalance and differences in comorbidity profiles between healthy controls and patients with OLP preclude attributing these differences to OLP alone. Within the two OLP groups, which were similar in age, the erosive group consistently showed descriptively higher OHIP‐G14 scores than the reticular group, although none of these subtype differences reached statistical significance.

Our findings are consistent with previous studies demonstrating the suitability of the OHIP‐14 for capturing patient‐perceived burden in OLP (Hegarty et al. 2002). Previous reports have described greater OHRQoL impairment in erosive‐ulcerative OLP, and the descriptively higher OHIP‐G14 scores observed in the erosive group of our cohort are directionally consistent with these findings (Daume et al. 2020). However, the absence of statistically significant differences between the OLP subtypes precludes confirmation of a subtype‐specific effect. The pattern of high scores for physical pain and psychological discomfort is also consistent with previous reports (Daume et al. 2020; Stolte et al. 2024). A systematic review and meta‐analysis further support the robustness of OHRQoL impairment in OLP and highlights pain‐related domains as key drivers of this burden (Yuwanati et al. 2021).

The simultaneous assessment of oral‐specific and generic patient‐reported outcome measures (PROMs) represents a strength of the present study by enabling comparison between oral disease burden and broader psychological well‐being. Although studies combining oral‐specific and psychological measures in OLP have been published previously, considerable heterogeneity in PROM selection has limited comparability across studies (Wiriyakijja et al. 2018). In this context, our study contributes a clinically pragmatic assessment battery combining OHIP‐G14 with PSS‐10 and WHO‐5. While PSS‐10 has been validated for use in OLP cohorts, the WHO‐5, to the authors' best knowledge, has not previously been reported alongside the OHIP within the same OLP cohort. This combination therefore provides complementary information by contrasting oral‐specific impairment with broader aspects of psychological well‐being (Heun et al. 2001; Topp et al. 2015; Wiriyakijja et al. 2018).

Despite pronounced oral‐specific impacts captured by OHIP‐G14, neither perceived stress nor general well‐being differed between groups. Within the limitations of this exploratory study, the absence of differences in PSS‐10 and WHO‐5 suggests that burden in this cohort was predominantly oral‐condition specific rather than reflecting generalized psychosocial distress. This finding is not necessarily contradictory, as OHIP‐G14 captures disease‐proximal impairment, whereas PSS‐10 and WHO‐5 assess broader constructs that may be influenced by contextual factors, disease duration, coping resources, comorbidity, and symptom activity at the time of assessment (Cerqueira et al. 2018). Similarly, 20%–30% of participants in each group scored below the commonly used WHO‐5 screening threshold, suggesting that reduced well‐being was not specific to OLP in the present cohort.

From a clinical and research perspective, our findings support the use of oral‐specific PROMs such as the OHIP‐G14 for assessing patient‐perceived burden in OLP. Generic instruments including PSS‐10 and WHO‐5 may provide complementary information on broader psychosocial health but appeared less sensitive for distinguishing OLP‐related burden in this cohort. These findings support the inclusion of oral‐specific PROMs when assessing patient‐perceived disease burden and may help guide outcome selection in future clinical studies and routine care. This interpretation is consistent with the broader emphasis on selecting PROMs according to the target construct and intended application in clinical care and trials (Prinsen et al. 2016, 2018).

### 4.1 Limitations

Several limitations should be considered. Healthy controls were younger and had fewer comorbidities than patients with OLP. Because age distributions showed limited overlap, post‐hoc covariate adjustment was not considered methodologically reliable. Consequently, the comparisons between controls and patients should be interpreted as unadjusted associations. Importantly, the reticular and erosive OLP groups were similar in age, making the exploratory within‐OLP comparison less affected by this source of confounding. However, this exploratory pilot study was not powered to detect differences between OLP subtypes, and the absence of significant subtype differences should therefore not be interpreted as evidence of equivalence. Nevertheless, the study provides data that may inform sample size calculations for future confirmatory studies.

The cross‐sectional design precludes causal inference, and potential confounders, including disease duration, lesion extent, pain severity, socioeconomic status, educational level, marital status, psychosocial coping resources, and objective measures of periodontal status, were not assessed. Medication‐related confounding cannot be excluded, as previous therapy was not systematically reported and antidepressant use was documented but not excluded. However, antidepressant use was not reported in the erosive OLP group and is therefore unlikely to explain the higher descriptive OHIP‐G14 burden in this subgroup.

Selection bias cannot be excluded because participants were recruited from a specialized university outpatient clinic, which may overrepresent patients with more severe or persistent disease. Finally, self‐reported outcomes remain susceptible to response bias.

## 5 Conclusions

In this cohort, patients with OLP reported substantially poorer OHRQoL across the OHIP‐G14 total score and all subdomains, indicating that OHIP‐G14 captures relevant aspects of patient‐perceived oral health burden in OLP. Erosive OLP showed the highest descriptive burden, particularly in pain‐ and function‐related dimensions. In contrast, PSS‐10 and WHO‐5 did not differ significantly between groups, indicating that oral‐specific and generic patient‐reported measures captured different aspects of patient experience in this cohort. These findings support the use of oral‐specific patient‐reported outcomes such as OHIP‐G14 for characterizing patient‐perceived burden in OLP, while broader psychosocial instruments may serve as complementary measures when clinically indicated.

## Author Contributions

Conceptualization: Kim N. Stolte and Henrik Dommisch. Methodology: Kim N. Stolte. Formal analysis: Sumru Sinem Dogan. Investigation: Kim N. Stolte. Data curation: Sumru Sinem Dogan and Kim N. Stolte. Writing—original draft preparation: Kim N. Stolte and Sumru Sinem Dogan. Writing—review and editing: Henrik Dommisch and Sameh Attia. Visualization: Kim N. Stolte and Sumru Sinem Dogan. All authors have read and agreed to the published version of the manuscript.

## Ethics Statement

The study was approved by the Ethics Committee of Charité—Universitätsmedizin Berlin (EA4/244/23).

## Consent

Written informed consent was obtained from all participants prior to inclusion. The study is reported in accordance with the STROBE (Strengthening the Reporting of Observational Studies in Epidemiology) guidelines for observational studies.

## Conflicts of Interest

The authors declare no conflicts of interest.

## Supporting information


Supporting File 1

Supporting File 2

## Data Availability

The data are available from the corresponding author upon reasonable request, subject to applicable ethical approval and data protection regulations.
