# Supplementary material for: Oral Health‐Related Quality of Life, Perceived Stress, and Well‐Being in Oral Lichen Planus Subtypes
Source: Clin Exp Dent Res. 2026 Nov 9;12(6):e70468. doi: 10.1002/cre2.70468 (PMC13747201; doi:10.1002/cre2.70468)
Supplement: Supplementary file 1 [file CRE2-12-e70468-s001.docx]

Supplementary Material

**Oral Health-Related Quality of Life, Perceived Stress and Well-Being in Oral Lichen Planus Subtypes**

Kim N. Stolte^1^, Sumru Sinem Dogan^1^, Sameh Attia^1^, Henrik Dommisch^1^

1 Department of Periodontology, Oral Medicine and Oral Surgery, Charité – Universitätsmedizin Berlin, Aßmannshauser Straße 4-6, 14197 Berlin, Germany

*****	Correspondence: kim-natalie.stolte@charite.de

Supplementary Material

Figure S1: Oral Health Impact Profile-14 (OHIP-G14) outcomes in the healthy controls versus oral lichen planus. Bars show mean ± standard deviation for each OHIP-G14 subdomain and the total score, comparing healthy controls with the pooled OLP cohort (reticular and erosive subtypes combined).

**Table S1:** OHIP-G14 Score differences (mean±SD) between healthy controls and patients with OLP (reticular and erosive subtype).

| **OHIP-G14 Subdomain** | **Healthy** | **OLP** | **p-value** |
| --- | --- | --- | --- |
| Functional limitation | 0.40 ± 1.05 | 1.25 ± 1.61 | 0.019 |
| Physical pain | 0.70 ± 1.90 | 4.03 ± 2.35 | <0.001 |
| Psychological discomfort | 0.75 ± 1.65 | 2.63 ± 2.54 | 0.002 |
| Physical disability | 0.35 ± 1.14 | 1.80 ± 2.07 | 0.001 |
| Psychological disability | 0.40 ± 1.19 | 1.65 ± 1.78 | 0.002 |
| Social disability | 0.05 ± 0.22 | 0.88 ± 1.40 | 0.010 |
| Handicap | 0.20 ± 0.62 | 0.95 ± 1.26 | 0.007 |
| **Total OHIP-G14 Score** | **2.85 ± 6.54** | **13.18 ± 10.01** | **<0.001** |

**Table S2:** Perceived Stress Scale (PSS) Score (mean±SD) in healthy controls, patients with reticular OLP and patients with erosive OLP.

| **PSS Subdomain** | **Healthy** | **Reticular OLP** | **Erosive OLP** | **p-value** |
| --- | --- | --- | --- | --- |
| PSS-H | 13.90 ± 4.23 | 14.65 ± 4.74 | 12.60 ± 4.20 | 0.335 |
| PSS-S | 8.60 ± 3.56 | 9.95 ± 2.70 | 9.90 ± 3.96 | 0.185 |
| **PSS Total Score** | **12.50 ± 5.58** | **14.60 ± 6.30** | **12.50 ± 6.18** | **0.450** |
